# Isotherms, kinetics and thermodynamic mechanism of methylene blue dye adsorption on synthesized activated carbon

**DOI:** 10.1038/s41598-023-50937-0

**Published:** 2024-01-10

**Authors:** Atef El Jery, Heba Saed Kariem Alawamleh, Mustafa Humam Sami, Hussein Abdullah Abbas, Saad Sh. Sammen, Amimul Ahsan, M. A. Imteaz, Abdallah Shanableh, Md. Shafiquzzaman, Haitham Osman, Nadhir Al-Ansari

**Affiliations:** 1 Department of Chemical Engineering, College of Engineering, King Khalid University, 61411 Abha, Saudi Arabia; 2 Department of Basic Scientific Sciences, Al-Huson College, Al-Balqa Applied University, P. O. Box 50, Al-Huson, 21510 Jordan; 3 Department of Pharmacy, Al-Noor University College, Nineveh, Iraq; 4 College of Nursing, National University of Science and Technology, Dhi Qar, Iraq; 5 Department of Civil Engineering, College of Engineering, University of Diyala, Baquba, Diyala Governorate 32001 Iraq; 6 Department of Civil and Environmental Engineering, Islamic University of Technology (IUT), Gazipur, 1704 Bangladesh; 7 Department of Civil and Construction Engineering, Swinburne University of Technology, Melbourne, Australia; 8 Research Institute of Sciences and Engineering, University of Sharjah, 27272 Sharjah, United Arab Emirates; 9 Department of Civil and Environmental Engineering, University of Sharjah, 27272 Sharjah, United Arab Emirates; 10 Department of Civil Engineering, College of Engineering, Qassim University, 51452 Buraidah, Saudi Arabia; 11 Civil, Environmental and Natural Resources Engineering, Lulea University of Technology, 97187 Lulea, Sweden

**Keywords:** Environmental sciences, Civil engineering

## Abstract

The treatment of methylene blue (MB) dye wastewater through the adsorption process has been a subject of extensive research. However, a comprehensive understanding of the thermodynamic aspects of dye solution adsorption is lacking. Previous studies have primarily focused on enhancing the adsorption capacity of methylene blue dye. This study aimed to develop an environmentally friendly and cost-effective method for treating methylene blue dye wastewater and to gain insights into the thermodynamics and kinetics of the adsorption process for optimization. An adsorbent with selective methylene blue dye adsorption capabilities was synthesized using rice straw as the precursor. Experimental studies were conducted to investigate the adsorption isotherms and models under various process conditions, aiming to bridge gaps in previous research and enhance the understanding of adsorption mechanisms. Several adsorption isotherm models, including Langmuir, Temkin, Freundlich, and Langmuir–Freundlich, were applied to theoretically describe the adsorption mechanism. Equilibrium thermodynamic results demonstrated that the calculated equilibrium adsorption capacity (*q*_*e*_) aligned well with the experimentally obtained data. These findings of the study provide valuable insights into the thermodynamics and kinetics of methylene blue dye adsorption, with potential applications beyond this specific dye type. The utilization of rice straw as an adsorbent material presents a novel and cost-effective approach for MB dye removal from wastewater.

## Introduction

The most significant problem that has arisen with the development of human society is environmental pollution^1,2^. Meanwhile, the presence of dye pollutants in textile wastewaters is of great concern due to their complex molecular structure and non-biodegradability^3^. Currently, wastewater from dye-related industries is recognized as a major environmental issue. For instance, approximately 51% of the dye produced enters the sewage during the dyeing process. Dye pollutants can adversely affect aquatic photosynthetic activities and, due to the presence of metals, aromatics, and other compounds in their structure, they can be toxic and harmful to the environment. Additionally, dyes can cause serious health issues in humans, such as liver, kidney, and central nervous system disorders. Mutagenicity, carcinogenesis, chromosome breakage, and respiratory problems are among the effects of excessive presence of dyes in the environment^4^.

However, treating industrial effluents containing dyes is challenging due to the stable nature of dye molecules and their resistance to aerobic digestion. Furthermore, the usual dye removal methods are economically undesirable or technically complicated because of the low concentration of dye molecules in industrial effluents^5^. To date, various methods such as membrane processes, absorption, coagulation, and advanced oxidation have been applied to remove dyes^6^.

Alipour et al.^7^ investigated the removal of basic violet 16 dye from wastewater using a magnetite graphene oxide nanocomposite (Fe3O4@GO) optimized by the response surface methodology based on the Box–Behnken method. The study highlights the potential of Fe3O4@GO as an efficient adsorbent for the removal of basic violet 16 dye from aqueous solutions.

Methylene blue (MB) is among the most commonly used chemicals in the cotton dyeing and wood industries; it is a dark green solid that may cause irritation of the respiratory tract, skin, and eye, in addition to cyanosis and bluish skin discoloration. However, tons of wastewater are discharged to the environment every year from different industrial sources such as printing, textiles^8^, plastics^9^, cosmetics^6,10^, leather^11^, and dye manufacturing industries^12^.

The removal of dyes from industrial effluents, especially from textile and dyeing factories, is an important measure to control pollution^13^. The presence of even a small amount of dye in water reduces the clarity and concentration of dissolved oxygen, posing a threat to aquatic life in the receiving environment. Air pollution and the environmental effects of industrial activities have been observed for decades^14^.

Several methods are used for treating methylene blue (MB) in wastewater, including adsorption, liquid–liquid extraction^15^, microbiological or enzymatic decomposition^16^, chemical oxidation^17^, and membrane filtration^18^. Among these, the adsorption method is the most commonly used due to its simplicity, low cost, and high operational life^19–21^. These methods can be categorized into biological, physical, and chemical processes. Biological methods are more economical than chemical and physical methods; biological degradation methods such as decolorization by fungi, surface adsorption, and other biological systems can collect and destroy various pollutants. These methods are used in purifying industrial wastes. However, the disadvantages of these methods include the need for a large space, sensitivity to environmental changes, such as toxicity and chemicals, and their high flexibility^22^.

Chemical processes include electric flotation, coagulation, and precipitation-flocculation. The accumulation of high concentrations of sludge, high cost, and excessive consumption of chemicals are some of the limitations of these methods^23^.

Various physical processes such as membrane filtration and surface adsorption processes have widely been applied to remove dyes. The limited life before sedimentation in the membrane and economic issues are limitations of membrane filtration processes. However, surface adsorption is the process of adsorbing spices in liquid or gas in contact with a solid surface. Surface adsorption begins with van der Waals forces as weak forces and ends with strong forces, such as ionic and metallic forces^24^.

Afshin et al.^25^ presented a novel approach for producing activated carbon from filamentous algae and magnetizing it with Fe3O4 for the efficient removal of cephalexin from aqueous media. The study highlights the potential of activated carbon as an effective adsorbent for the removal of pollutants from wastewater.

Among the various methods of dye removal, the adsorption of metal–organic frameworks (MOFs) have recently attracted considerable attention in research; metallurgical frameworks and magnetic nanocomposites have attracted the attention of researchers owing to their industrial applications in adsorption processes^26^. Magnetic particles are placed in the coordination polymers^27^, and magnetic MOFs are obtained from MOFs together with magnetic particles, which can be metal oxides, pure metals, or metal alloys^28^. Owing to the large area of the pore surfaces, design and engineering capability, possibility of separation, and reusability, they have recently been used as efficient adsorbents for dye treatment of textile wastes^29^. Moreover, magnetic ferrite nanoparticles in the bed of MOFs enter the intuition by inducing magnetic properties in the resulting nanocomposite^30^. Consequently, the resulting magnetic nanocomposite acts as a non-homogeneous adsorbent, which can be easily used to remove toxic and polluting dyes. Finally, it can be separated easily with an external magnet. In recent years, MOFs have been applied to the treatment of dyes from wastewater; however, they have not been widely accepted because of the possible risk of difficult and nonreusable separation^31^. Zhao et al.^32^ developed a magnetic MOF composite on graphene oxide sheets. They placed magnetic particles (SiO_2_@Fe_3_O_4_) on graphene oxide sheets (1-HKUST) using three-dimensional MOF modifications, and the synthesized nanocomposite MOF was used for insecticide adsorption in water. Zhao et al.^33^ synthesized and identified a magnetic (1-HKUST@Fe_3_O_4_) MOF using a co-precipitation method. They reported the treatment of MB dye using the synthesized MOF. Furthermore, Foong et al.^34^ performed a new catalytic reduction of MB dye using a novel synthesized composite (101-MIL@Fe_3_O_4_) MOF.

Among the methods that have been applied to remove pollutants, adsorption has attracted considerable attention. Currently, the use of adsorbent materials is among the most cost-effective and economical processes for removing pollution from the ground or polluted waters^35^. These materials can transfer the species from the liquid to the solid phase, which makes it possible to treat MB dye in water. Among the factors affecting the selection of adsorbent materials for the treatment of MB dye compounds, hydrophobicity, biodegradability, availability, high rate of adsorption, high capacity, and high physical and chemical resistance are significant.

Adsorbents are generally divided into three categories: natural organic, mineral, and synthetic adsorbents. Natural materials are relatively inexpensive, abundantly available, and have a very high adsorption capacity; aerogels, perlite, and mineral soils are among the most important mineral adsorbents^36^; polypropylene and polyurethane are the most common synthetic organic materials used for pollution treatment.

In recent years, many materials, such as agricultural residues, have been used to adsorb MB from wastewater^37^. Activated carbon, owing to its excellent adsorption ability, is one of the most widely applied substances for dye treatment in wastewater; however, because it is costly, many studies have been carried out to find an alternative^38^. In 2021, Jawad et al.^39^ introduced a new approach to produce a novel active carbon, using dragon fruit peel, to treat MB dye from a desired solution. Ighalo et al.^40^ studied the effect of the pore size on the kinetics of MB dye adsorption. However, despite many studies on activated carbon with different precursors, no in-depth research has been carried out to thermodynamically understand the mechanism of adsorption. Every year, millions of tons of straw are discarded globally as waste, which in addition to being environmentally unsustainable, destroys a huge source of precious materials^41,42^. Rice is among the globally cultivated crops, and Fathy et al.^43^ studied the effect of rice straw modification by alkali acid on MB dye treatment in binary and single solutions**.**

The production of straw-based adsorbents is a promising way to use this valuable bioresource for the adsorption of MB from industrial wastewater. The purpose of the present study was to evaluate the effects of operational conditions, including pH, temperature, and MB dye concentration, on the removal efficiency. Thus, three different models of adsorption isotherms, Langmuir, Temkin, and Freundlich models, which differ in their assumptions, nature of the adsorbent surface, and shape of the isotherm, were used to correlate the process of adsorption. In this study, an analytical isotherm expression was developed to simulate the effects of pH and temperature on surface adsorption. The proposed model, which is based on the Langmuir isotherm, is a suitable alternative for complex simulations that require numerical solution methods. It was shown that for the obtained experimental data, there is a linear correlation between the isotherm coefficients and pH or temperature.

Several studies correlate the surface adsorption isotherm equations with the experimental data at different pH values. Eswad-El et al.^44^ correlated the experimental data of the surface adsorption of Ni(II) and Cu(II) on humic acid adsorbents at different pH values using the Langmuir isotherm. Silber et al.^45^ obtained experimental data for the surface adsorption of Zn ions on two different types of adsorbents, assuming a constant pH during the adsorption process, and correlated the surface adsorption model with the developed Langmuir model. Youcef et al.^46^ applied the Langmuir isotherm model to study the adsorption of MB dye on Algerian palygorskite.

Mustafa et al.^47^ correlated the experimental data for cadmium ion adsorption at different pH values using the Freundlich isotherm. Arora et al.^48^ also investigated the removal of MB dye from wastewater using the Freundlich isotherm. Regelink et al.^49^ studied surface adsorption and precipitation of Ni ions. They compared the experimental data at five different pH values using different multilevel adsorption models. They also reported the amount of nickel ion adsorption with changes in pH. Han et al.^50^ correlated the experimental data obtained from the surface adsorption of uranium using the Langmuir and Redlich–Peterson models at different pH values.

Hu et al.^30^ fitted the experimental surface adsorption data at different pH values using the Langmuir and Freundlich isotherm models and obtained a linear relationship between the isotherm parameters and pH. They also presented a modified Langmuir–Freundlich relationship and measured its validity using the obtained experimental data, which suggested that this model can predict surface adsorption at different pH values.

The use of statistical physics models in the adsorption of dye molecules on bioadsorbents has been a topic of interest in recent years. Several studies have investigated the application of statistical physics models to understand the adsorption mechanism and optimize the process. In this literature review, we will discuss some of the recent studies that have used statistical physics models to investigate the adsorption of dye molecules on bioadsorbents.

Khalfaoui et al.^51^ used statistical physics modeling to investigate the adsorption of dyes on modified cotton. The study estimated all the mathematical parameters in the model, including the receptor site density and the half-saturation concentration. The results showed that the statistical physics model provided a good fit to the experimental data and could be used to optimize the adsorption process.

In another study, Raval et al.^52^ used statistical physics modeling to evaluate the adsorption properties of chitosan-zinc oxide nanocomposites for the removal of an anionic dye. The study demonstrated an effective synthesis route that could be implemented to prepare new adsorbents using chitosan at a large-scale for water treatment.

In a study by Hanafy et al.^53^, statistical physics modeling was used to interpret the adsorption of dye remazol black B on natural and carbonized biomasses. The study employed natural and carbonized pine-fruit shells to study the adsorption of remazol black B dye from aqueous solution. The results showed that the statistical physics model could be used to predict the adsorption capacity of the adsorbent and optimize the process.

In a recent study, Zhang et al.^54^ established an advanced statistical physics adsorption model to investigate the adsorption of Congo Red and Methylene Blue onto nanopore-structured Ashitaba waste and walnut shell-based activated carbons. The study demonstrated that the statistical physics model could provide a good fit to the experimental data and could be used to optimize the adsorption process.

Finally, in a study by Sangon et al.^55^, waste rice straw was used to produce highly effective carbon-based adsorbents for dyes removal. The pre-carbonized potassium hydroxide activated carbon was demonstrated to be highly effective at the adsorption of methylene blue and Congo red dyes. The study provides valuable insights into the use of waste materials for the production of effective adsorbents.

In this study, using a cheap material (i.e. rice straw) and a simple and economical method, an adsorbent that is selective and robust in adsorbing MB has been produced. In addition to the experimental study of MB dye adsorption on the fabricated adsorbent, the adsorption isotherms were investigated under different process conditions (e.g. temperature and pH). The goal of previously published papers in this field was to adsorb MB dye, whereas this study aims to optimize the process while understanding the thermodynamics and kinetics of the process.

## Materials and methods

### Materials and chemicals

Rice straw was collected from irrigated and rain-fed fields in Jordan. Permission to collect rice straw was obtained from the relevant authorities. Experimental research and field studies on plants, including the collection of plant material, comply with relevant institutional, national, and international guidelines and legislation. Sulfuric acid (H_2_SO_4_) with purity of 99.999% and reagent grade anhydrous potassium hydroxide (KOH) pellets (≥ 98% purity) were obtained from Sigma-Aldrich. MB was also purchased from Sigma-Aldrich, and all chemical reagents were of analytical grade. The rice straw was cut into 0.5-cm pieces and dried at 90 °C.

### Characterization

Scanning Electron Microscopy (SEM, Thermo Fisher, VolumeScope 2) was performed to characterize the morphology and structure of the pores on the rice straw surface. Moreover, Energy Dispersive X-ray Spectroscopy (EDX, Hitachi, Quantax75) was applied to detect the elemental content on the adsorbent surface, and the functional groups of the adsorbent surface were analyzed using Fourier transform infrared (FTIR, JASCO FT/IR-4X) analysis over the wavenumber range of 500–4000 cm^−1^.

### Experimental procedure

A novel and simple procedure was used to fabricate a low-cost adsorbent from rice straw. First, the rice straw was dried and soaked in an H_2_SO_4_ solution (0.1 M) for 24 h, washed using deionized water, and dried overnight at 95 °C. Subsequently, the dried substances were soaked in KOH (0.2 M) for 24 h at 25 °C; it was then washed with deionized water and dried at 95 °C for 24 h.

To prepare synthetic wastewater, solutions were prepared using an aqueous solution of MB dye. All experiments were performed in a 5-L flask at different pH and temperature values; pH adjustment of the solutions to the desired value was carried out using H_2_SO_4_ or NaOH.

To calculate the thermodynamic adsorption isotherm parameters, the equilibrium adsorption process was investigated at different pH values (3–9) and temperatures (293–333 K). The adsorbent material was added to water, pH of the mixture was adjusted, and it was allowed to stand for approximately 30 min. The pH was measured using a pH meter (Milwaukee, MW802, Germany) before and after adsorption. Subsequently, different volumes of 4 mg/L MB dye solution were added to the adsorbent system. Finally, the mixtures were stirred for 20 h to reach equilibrium, and all the experiments were performed several times.

To analyze the efficiency of the treatment process, the concentration of the MB dye was measured at a wavelength of 664 nm using a Shimadzu 2570 UV–visible spectrophotometer. The adsorption capacity of MB was calculated using Eq. (1), where C_0_ (mg/L) and C_e_ (mg/L) are the initial and equilibrium concentration of the adsorbed dye, respectively, V is the MB dye solution volume in liters, and m is the dry weight of rice straw in grams.

1 $$Q = \frac{{\left( {C_{0} - C_{e} } \right)V}}{m}$$

The adsorption of the control group can be measured as the difference between the total MB amount and residual MB. HCl was applied to desorb MB at the end of the experiment. Therefore, the adsorption amount can be evaluated using Eq. (2), where *C*_*d*_ (mg/L) is the MB concentration, *V*_*d*_ is the volume of the dye solution, and *m*_*d*_ is the dry weight of the rice straw. The adsorbed amount of MB dye was obtained as the difference between the total treatment amount and adsorbed amount after removal.

2 $$Q_{d} = \frac{{C_{d} V_{d} }}{{m_{d} }}$$

The concentration equilibrium relationship in the system was investigated using adsorption isotherms at a constant temperature. The maximum adsorption capacity was evaluated using equilibrium adsorption isotherms. Thus, three different adsorption isotherm models were used in this study, Langmuir, Temkin, and Freundlich isotherms, which differ in their assumptions, nature of the adsorbent surface, and shape of the isotherm.

Similar methods have also been used by other researchers to perform adsorption isotherms at different pH values^44,45,47,48^**.**

The Langmuir isotherm model^56^ is applied to explain the nonlinear adsorption and proposes that take place on a homogeneous surface without interactions between the adsorbed species. In this model, each binding position accepts only one molecule and has the same energy. The Langmuir isotherm model is given by Eq. (3), which is in a linear form.

3 $$\frac{{C_{e} }}{{q_{e} }} = \frac{{C_{e} }}{{q_{m} }} + \frac{1}{{K_{l} q_{m} }}$$

where *q*_*e*_ and *C*_*e*_ are the adsorption capacities at the equilibrium and equilibrium concentrations, respectively; *K*_*l*_ and *q*_*m*_ are the Langmuir constant and maximum adsorption capacity, respectively. According to Eq. (3), the Langmuir giving correlation coefficients (*R*^2^) validity can be confirmed using a linear graph of $\frac{{C_{e} }}{{q_{e} }}$ versus C_e_. The reversible adsorption on different adsorption energy sites can be reported using Freundlich isotherm model as an empirical equation.

Freundlich equation describes the adsorption of a solute from a source to solid surface; it assumes that stronger binding sites are occupied first, and other sites are occupied in the decreasing order of energy. This model presents monolayer adsorption with an inhomogeneous energy distribution in the active sites. The Freundlich isotherm model, in linear form, is shown in Eq. (4), where *K*_*F*_ is the Freundlich constant, and *n* is related to the adsorption intensity and called the heterogeneity factor.

4 $$\ln q_{e} = \frac{{\ln C_{e} }}{n} - \ln K_{F}$$

The third isotherm model is the Temkin isotherm model, which is described in Eq. (5), where T is the temperature, *K*_*T*_ and *b* are adjustable constants, and *R* is the universal gas constant.

5 $$q_{e} = \frac{RT}{b}\left( {\ln C_{e} + \ln K_{T} } \right)$$

## Results and discussion

**Figure 1:**
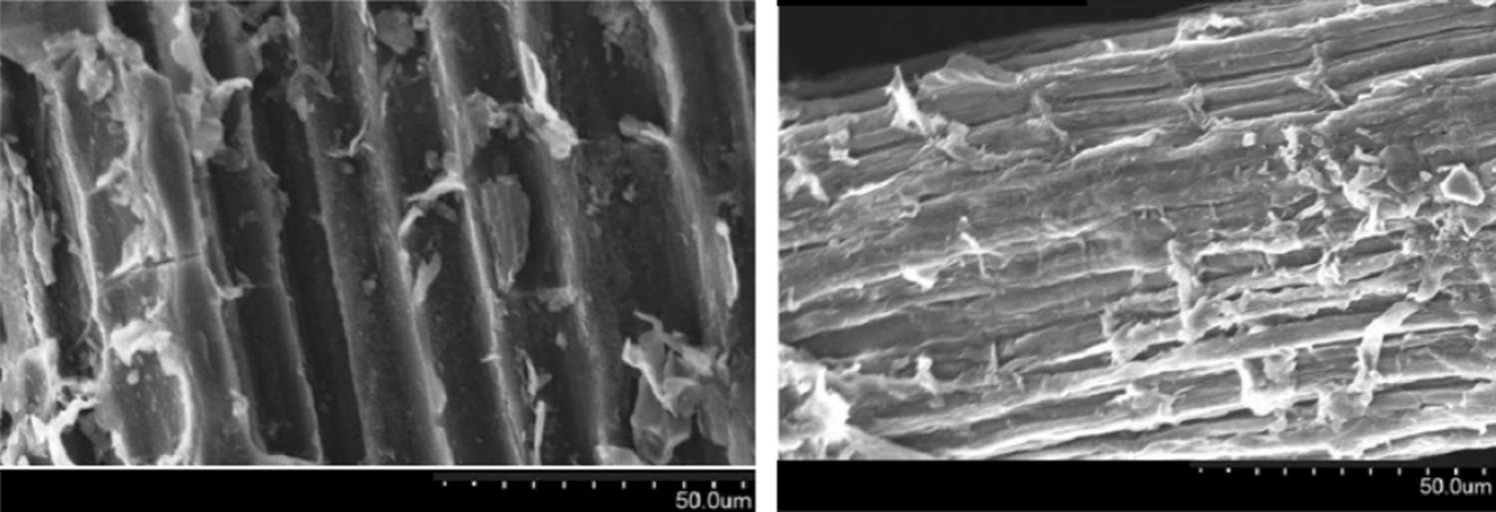
SEM image of rice straw adsorbent with ×500  magnification.

An SEM image of the rice straw adsorbent is shown in Fig. 1. As can be seen from the SEM analysis, the surface pores of the adsorbent were reasonably uniform in the range of 45–55 μm, which caused dye adsorption.

Table 1 shows the contents of the different elements on the adsorbent surface, which were detected via EDX analysis. The adsorbent surface is dominated by C, Ca, O, Si, which were involved in the adsorption experiments, with values of 47.7, 1.6, 41.7, and 8.7 wt.%, respectively.

**Table 1:** EDX study of rice straw adsorbent.

Element	Apparent concentration	Weight % (%)	Atomic % (%)
C	11.93	47.7	54.7
O	20.22	41.7	37.2
Si	5.10	8.7	4.4
Ca	0.91	1.6	0.6

**Figure 2:**
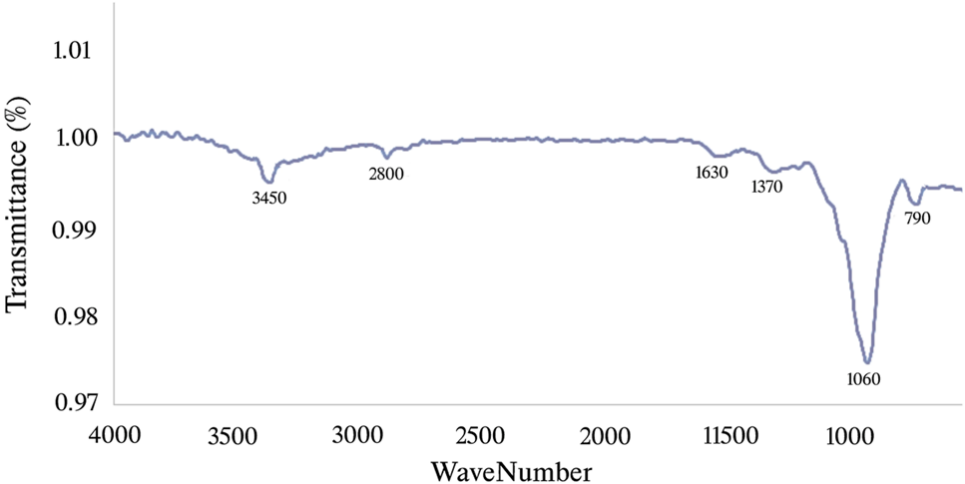
FTIR results of the rice straw absorbent.

Figure 2 shows the functional group identification of the manufactured adsorbent, and the FTIR results are summarized in Table 2. The peak at 1060 cm^−1^ is attributed to the C–O group. The absorbance bands at 1370 cm^−1^ and 1630 cm^−1^ are assigned to the C–C and C=O groups, respectively; the peaks at 2800 cm^−1^ and 3450 cm^−1^ are related to C–OH and O–H groups, respectively.

**Table 2:** Functional groups of the rice straw adsorbent.

Peak number	Frequency (cm^−1^)	Functional groups	Type of bond	Intensity
1	790	Si–O		
2	1060	C–O	Stretching	Strong
3	1370	C–C		Weak
4	1630	C=O	Stretching	Strong
5	2800	C–OH	Stretching	Weak
6	3450	OH		

**Figure 3:**
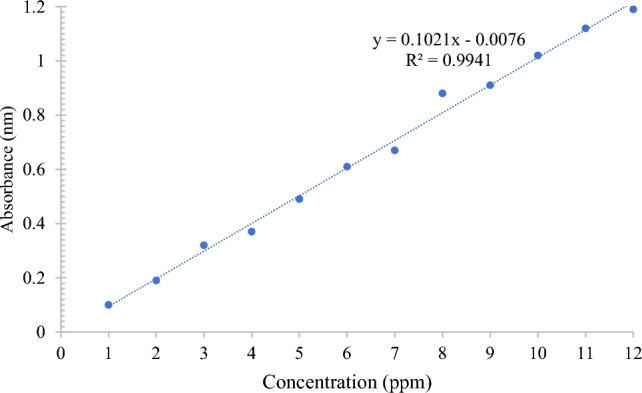
Calibration curve of MB dye.

To obtain various densities at different MB dye concentrations in the solution, the calibration curve for the MB dye was plotted in terms of concentration in ppm vs. wavelength reading, and the k factor was calculated as shown in Fig. 3.

**Figure 4:**
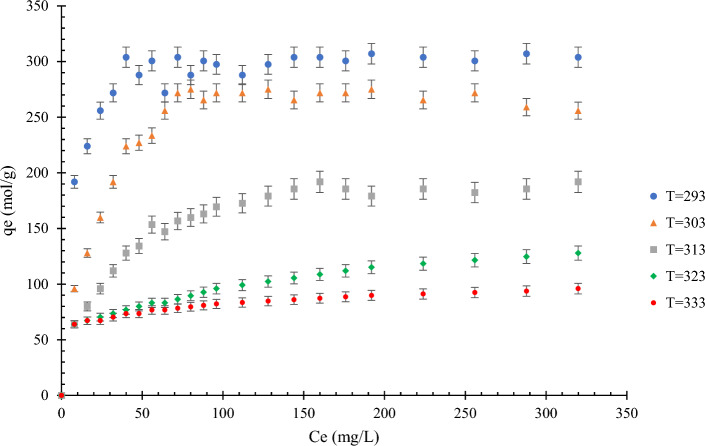
Effect of temperature on the MB dye adsorption on modified rice straw at different temperatures (293–333 K) and pH  6; the amount of adsorbent = 1 g in 300 mL methylene blue.

**Figure 5:**
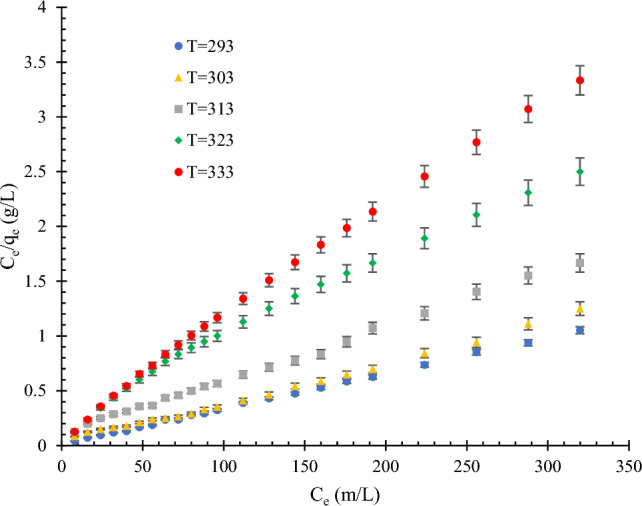
Linearized model of Langmuir isotherm at different temperatures (293–333 K) and pH  6; the amount of adsorbent = 1 g in 300 mL methylene blue.

Figure 4 shows the effects of temperature on the adsorption of MB dye. As shown in Fig. 4, a decrease in adsorption can be seen with increasing temperature, which can be attributed to MB adsorption being an exothermic process. The linearized model of the Langmuir isotherm at different temperatures is shown in Fig. 5. In all the experiments, the initial solution concentration of MB dye was 4 mg/L, and the amount of adsorbent was 1 g in 300 mL based on the MB dye concentration.

To correlate the adsorption process using isotherm models, three different adsorption isotherm models, Langmuir, Temkin, and Freundlich isotherms, were applied, and the isotherm parameters were determined using linearized experimental data. The measured parameters of the isotherms for MB dye treatment using modified rice straw at various temperatures are listed in Table 3.

**Table 3:** Parameters of isotherms for methylene blue dye treatment using modified rice straw at different temperatures (293–333 K) and pH 6.

Model	Parameters	293 K	303 K	313 K	323 K	333 K
Langmuir	*K*_*l*_ × 10^–4^	7.663	4.404	1.490	1.147	2.491
	*q*_*m*_ × 10^4^	9.64	8.58	6.36	4.14	3.01
	*R*^2^	0.999	0.994	0.997	0.986	0.997
Freundlich	*K*_*F*_ × 10^–2^	4.952	1.795	1.888	5.911	14.881
	*n*	10.1	4.05	3.39	4.69	8.41
	*R*^2^	0.683	0.731	0.898	0.965	0.980
Temkin	*K*_*T*_ × 10^–8^	4.851	1.784	0.012	0.0062	0.173
	*b* × 10^–7^	3.045	0.251	2.546	4.475	9.228
	*R*^2^	0.702	0.754	0.935	0.932	0.968

Adsorption isotherm constants can determine the characteristics of the surface, attraction of the synthesized adsorbent, and capacity for pollutant adsorption. The EDX analysis reveals that the adsorbent contained different surface functional groups that could be affected by the pH value in terms of adsorbing the substances.

**Figure 6:**
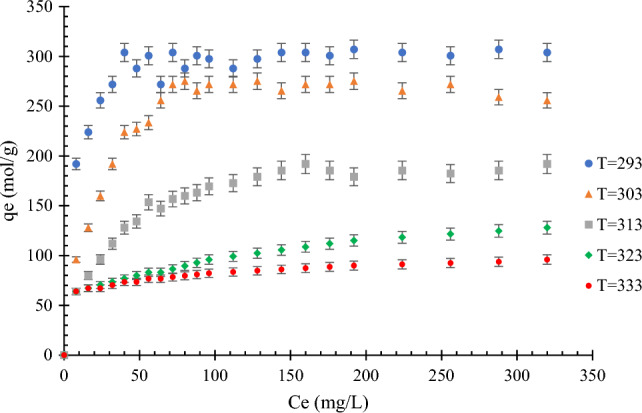
Effect of pH on the MB dye adsorption on modified rice straw at different pH values (2–10) and T = 303 K; the amount of adsorbent = 1 g in 300 mL methylene blue.

**Figure 7:**
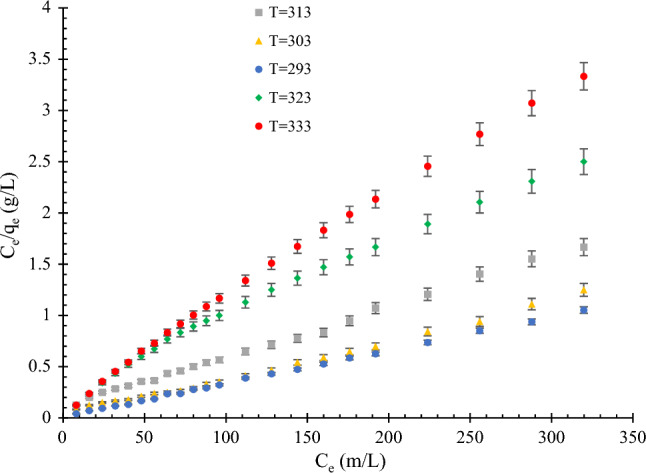
Linearized form of the Langmuir isotherm model at different pH values (2–10) and T = 303 K; the amount of adsorbent = 1 g in 300 mL methylene blue.

Clearly, with an increase in the pH value, the amount of hydroxyl (OH^-^) in the system increases and surface charge of the adsorbent particles becomes more negative; therefore, MB adsorption on the adsorbent is predicted to increase. However, the pH domain is among the key characteristics of wastewater, which can affect the surface characteristics of the adsorbent by changing the ionization degree. The results showed that at acidic pH values, owing to the protonation of active sites and an increase in the charge density, the removal efficiency of MB dye is lower; at alkaline pH values, the electrostatic attraction between the ions and dye is observed. Figure 6 shows the effect of pH on the adsorption of MB dye. As shown in Fig. 6, an increase in pH causes an increase in adsorption, which can be attributed to the bonding between the MB and rice straw adsorbent being disrupted by H^+^ ions at higher acidity. The linearized model of the Langmuir isotherm for different acidities is shown in Fig. 7.

To correlate the adsorption process using isotherm models, three different adsorption isotherm models, Langmuir, Temkin, and Freundlich isotherms, were used, and the isotherm parameters were determined by linearizing the experimental data. The isotherm parameters are listed in Table 4.

**Table 4:** Parameters of isotherm for methylene blue dye treatment using modified rice straw at different pH values (2–10) and T = 303 K.

Model	Parameters	pH 2	pH 4	pH 6	pH 8	pH 10
Langmuir	*K*_*l*_ × 10^–4^	0.998	1.490	4.404	7.663	43.583
	*q*_*m*_ × 10^4^	4.22	6.36	8.58	9.665	9.56
	*R*^2^	0.987	0.997	0.994	0.999	0.999
Freundlich	*K*_*F*_ × 10^–2^	5.911	1.888	1.795	4.951	6.515
	*n*	4.49	3.39	4.05	10.2	16.36
	*R*^2^	0.965	0.898	0.731	0.684	0.559
Temkin	*K*_*T*_ × 10^–8^	0.006	0.012	1.784	4.852	14.462
	*b* × 10^–7^	4.198	2.519	0.251	3.046	5.038
	*R*^2^	0.932	0.935	0.754	0.701	0.558

Because the Langmuir and Freundlich models both correlated well with the experimental adsorption data, the combined Langmuir–Freundlich model was used. Therefore, in the present work, the Langmuir–Freundlich isotherm was also applied to correlate the obtained data. The Langmuir–Freundlich equation, also known as the SIP equation, is a multidimensional equation that can predict the behavior of both Langmuir and Freundlich isotherms^57^.

Some studies have reported the adsorption capacity of adsorbent materials at different pH values and temperatures. For example, Xu et al.^58^ reported experimental Pb (II) adsorption data on an MX-80 bentonite adsorbent at different pH values. Regelink et al.^49^ reported Ni adsorption on a specific type of adsorbent at different pH values. They also compared their experimental data with the adsorption values predicted by a multilevel adsorption model.

**Figure 8:**
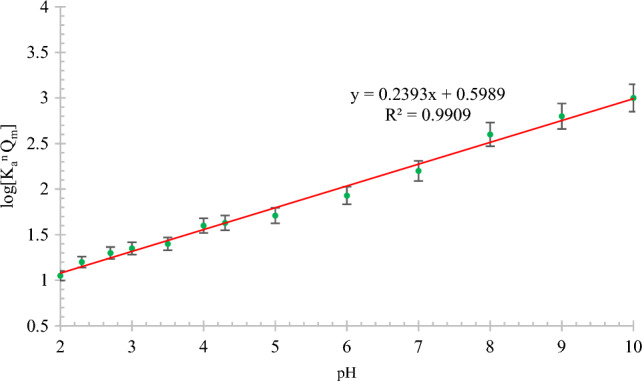
Relationship between pH and Langmuir–Freundlich equation constants for methylene blue dye adsorption on modified rice straw adsorbent.

Figure 8 shows the relationship between the Langmuir and Freundlich isotherm constants and pH for MB dye removal using the modified rice straw adsorbent. As shown in Fig. 8, a linear relationship with pH was obtained. With an increase in the pH value, the equilibrium coefficient in the Langmuir–Freundlich relationship increases linearly.

**Figure 9:**
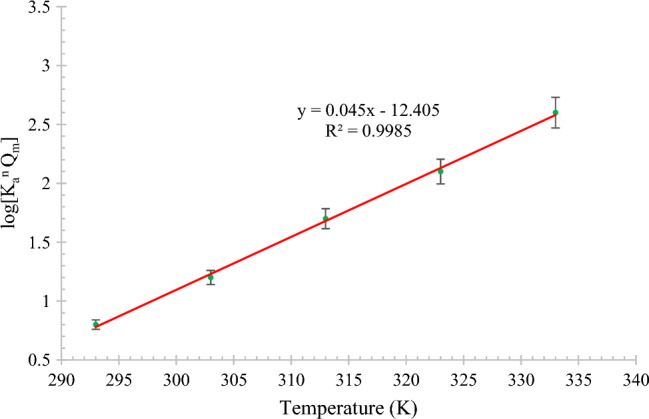
Relationship between temperature and Langmuir–Freundlich equation constants for methylene blue dye adsorption on modified rice straw adsorbent.

Figure 9 shows the relationship between the Langmuir and Freundlich isotherm constants and temperature for MB dye removal using the modified rice straw adsorbent. As shown in Fig. 9, a linear relationship with temperature was obtained; with an increase in temperature, the equilibrium coefficient in the Langmuir–Freundlich relationship increases linearly. The constants of the Langmuir–Freundlich relationship at various temperatures in presence of a similar adsorbent show a behavior similar to that at different pH values.

### Adsorption thermodynamics

The free energy of adsorption can be obtained using Eq. (6), where *K*_*D*_ can be evaluated at *q*_*e*_ = 0 in the *q*_*e*_/*C*_*e*_ vs. *q*_*e*_ plot.

6 $$\Delta G = - RT\ln K_{D}$$

Therefore, ΔH and ΔS, as the thermochemical parameters, can be calculated by applying the van’t Hoff equation (Eq. 7).

7 $$\ln K_{D} = - \frac{\Delta H}{{RT}} + \frac{\Delta S}{R}$$

The free energy, ΔG, of rice straw adsorption of MB is shown in Table 5. The calculated free energy values of MB adsorption on rice straw are negative, which indicates that the adsorption process can be carried out spontaneously. These results have been previously confirmed by other researchers^59,60^.

**Table 5:** Free energy, ΔG, of methylene blue adsorption on rice straw at various temperatures and pH  6.

T (K)	K_D_ (g/L)	ΔG (kJ/mol)
293	54.753	−9.751
303	15.464	−6.899
313	9.0864	−5.743
323	7.6151	−5.452
333	9.6774	−6.284

To analyze the obtained results, the free energy, ΔG, of MB adsorption on rice straw must be obtained; it is obtained using Eq. (6), and the results are listed in Table 5. To obtain ΔH and ΔS as the thermochemical parameters, Eq. (7), which is known as the van't Hoff equation, is used. Using Eq. (7), ΔH/R is −6167 kg K/mol and ΔS/R is equal to −17.3 kg/mol.

## Conclusions

In recent years, various materials, including agricultural residues, have been explored for MB dye adsorption from wastewater. However, a comprehensive thermodynamic understanding of the adsorption mechanism has been lacking. This study introduced the use of rice straw for the novel and cost-effective removal of MB dye from wastewater. The investigation of different operational conditions, such as temperature, pH, and MB dye concentration, aimed to optimize the adsorption process's removal efficiency. The results indicated that this novel adsorption method is strongly pH- and temperature-dependent and effective for treating MB dye in aqueous wastewater. The study also explored the equilibrium thermodynamic models of adsorption using various isotherm models, revealing the impact of pH and temperature variations on the constants of different isotherm models. In this study, the equilibrium thermodynamic models of adsorption were also investigated using three different adsorption isotherm models, namely, Langmuir, Temkin, and Freundlich models; moreover, Langmuir–Freundlich model was used to correlate the adsorption results. The results show that the constants of different isotherm models can change significantly by varying the pH and temperature values. A decrease in adsorption can be observed owing to an increase in the temperature, which can be attributed to the fact that MB adsorption is an exothermic process; an increase in pH causes an increase in adsorption, which can be attributed to the fact that, at higher acidity, the bonding between the MB and rice straw as an adsorbent can disrupted by H^+^ ions. The findings contribute to a deeper understanding of the adsorption process and its potential applications in wastewater treatment.

## Data Availability

The data that support the findings of this study are available on request from the corresponding authors.
