# Publisher Correction: Isotherms, kinetics and thermodynamic mechanism of methylene blue dye adsorption on synthesized activated carbon

**DOI:** 10.1038/s41598-024-55385-y

**Published:** 2024-02-27

**Authors:** Atef El Jery, Heba Saed Kariem Alawamleh, Mustafa Humam Sami, Hussein Abdullah Abbas, Saad Sh. Sammen, Amimul Ahsan, M. A. Imteaz, Abdallah Shanableh, Md. Shafiquzzaman, Haitham Osman, Nadhir Al-Ansari

**Affiliations:** 1 Department of Chemical Engineering, College of Engineering, King Khalid University, 61411 Abha, Saudi Arabia; 2 Department of Basic Scientific Sciences, Al-Huson College, Al-Balqa Applied University, P. O. Box 50, Al-Huson, 21510 Jordan; 3 Department of Pharmacy, Al-Noor University College, Nineveh, Iraq; 4 College of Nursing, National University of Science and Technology, Dhi Qar, Iraq; 5 Department of Civil Engineering, College of Engineering, University of Diyala, Baquba, Diyala Governorate 32001 Iraq; 6 Department of Civil and Environmental Engineering, Islamic University of Technology (IUT), Gazipur, 1704 Bangladesh; 7 Department of Civil and Construction Engineering, Swinburne University of Technology, Melbourne, Australia; 8 Research Institute of Sciences and Engineering, University of Sharjah, 27272 Sharjah, United Arab Emirates; 9 Department of Civil and Environmental Engineering, University of Sharjah, 27272 Sharjah, United Arab Emirates; 10 Department of Civil Engineering, College of Engineering, Qassim University, 51452 Buraidah, Saudi Arabia; 11 Civil, Environmental and Natural Resources Engineering, Lulea University of Technology, 97187 Lulea, Sweden

Correction to: *Scientific Reports* 10.1038/s41598-023-50937-0, published online 10 January 2024

In the original version of this Article, Amimul Ahsan was omitted as a co-corresponding author. The corresponding authors for this Article are Amimul Ahsan and Nadhir Al-Ansari. Correspondence and request for materials should be addressed to ahsan.iut2@gmail.com and nadhir.alansari@ltu.se.

The original Article has been corrected.

